# *svapls*: an R package to correct for hidden factors of variability in gene expression studies

**DOI:** 10.1186/1471-2105-14-236

**Published:** 2013-07-24

**Authors:** Sutirtha Chakraborty, Somnath Datta, Susmita Datta

**Affiliations:** 1 Department of Bioinformatics and Biostatistics, University of Louisville, Louisville, KY-40202, USA

## Abstract

**Background:**

Hidden variability is a fundamentally important issue in the context of gene expression studies. Collected tissue samples may have a wide variety of hidden effects that may alter their transcriptional landscape significantly. As a result their actual differential expression pattern can be potentially distorted, leading to inaccurate results from a genome-wide testing for the important transcripts.

**Results:**

We present an R package svapls that can be used to identify several types of unknown sample-specific sources of heterogeneity in a gene expression study and adjust for them in order to provide a more accurate inference on the original expression pattern of the genes over different varieties of samples. The proposed method implements Partial Least Squares regression to extract the hidden signals of sample-specific heterogeneity in the data and uses them to find the genes that are actually correlated with the phenotype of interest. We also compare our package with three other popular softwares for testing differential gene expression along with a detailed illustration on the widely popular Golub dataset. Results from the sensitivity analyes on simulated data with widely different hidden variation patterns reveal the improved detection power of our R package compared to the other softwares along with reasonably smaller error rates. Application on the real-life dataset exhibits the efficacy of the R package in detecting potential batch effects from the dataset.

**Conclusions:**

Overall, Our R package provides the user with a simplified framework for analyzing gene expression data with a wide range of hidden variation patterns and delivering a differential gene expression analysis with substantially improved power and accuracy.

The R package svapls is freely available at http://cran.r-project.org/web/packages/svapls/index.html.

## Background

Several types of subject/sample specific factors constitute an important but often overlooked source of hidden variability in differential gene expression analyses. In a wide variety of situations these factors are triggered from certain specific biological, environmental or demographic profiles of the subjects corresponding to the collected tissue samples. The latent effects from these hidden factors can generate spurious signals of heterogeneity that may significantly distort the original differential expression pattern of the genes. In this context, a simple example is provided by the widely known batch-effect in microarray analyses, where subject tissue samples collected in separate batches can produce an additional effect of residual variation. This effect is still manageable as composition of the batches are known prior to analyses. But, numerous other factors may still exist that are not detectable from outside, but can potentially affect the subject-specific expression levels of the genes in different ways. They can in turn lead to complex latent expression structures in the entire genomic landscape of the data (e.g., confounded signals between the two groups of samples, correlated expression signals corresponding to a specific group of genes and samples affected by the hidden factors, etc.). The contributed impact of these factors, either acting singly or in consort can induce serious problems in multiple testing of differential expression for the genes. Thus, a number of truly significant genes can pass out undetected while many others may be wrongly flagged as positives. The consequence is a severe reduction in power (sensitivity) of the testing procedure accompanied by a substantially high rate of erroneous discoveries. Most of available softwares for differential gene expression analyses either overlook this broadly general issue of hidden variability or consider simple parametric regression approaches (linear regression, mixed effects models, etc.) to address the maladies of residual heterogeneity. However the complexity of problem necessitates the development of a more generalized and efficient technique that can identify these latent effects of variation in the data and adjust for them in order to deliver a more powerful and accurate inference on the actual expression pattern of the genes. This motivated us to construct a methodology [1] that provides an unified framework for handling these widely different types of spurious variability in the data.

We have built an R software **svapls** that uses the multivariate Non-Linear Iterative Partial Least Squares (NIPALS) algorithm [2] to extract the latent, unwanted effects of variation in a gene expression data and uses them to build an optimal ANCOVA model for detecting the truly differentially expressed genes between two types of samples/tissues. In the next section we describe the important functions in our package along with illustrative examples that explain their practical usage in detail. The following section ‘Comparative evaluation with other available software packages’ demonstrates its comparatively superior performance with respect to three other popular softwares: **sam**[3], **limma**[4] and **sva**[5] through a sensitivity analysis of two simulated differential gene expression datasets affected by complicated hidden variation patterns. Section ‘Application on the Golub data’ elucidates an application on a real-life dataset that proves the worth of our software through the adjustment for batch effects and detection of some additional phenotype-related genes that are deemed to be significant from their annotations in the literature. The manuscript ends with a discussion in Section ‘Discussion’.

## Implementation

### Brief overview of the package

This R package consists of the three primary functions: **fitModel**, **svpls** and **hfp**. Below we give a brief outline of them. The function applications are demonstrated on a simulated dataset affected by hidden variation (**hidden_fac.dat**) that is inbuilt as a part of the R package. 

•The first function **fitModel** fits an ANCOVA model to the original log-transformed gene expression data, with a certain number of PLS scores as surrogate variables (specified by **n.surr**) or the simple ANOVA model [6,7] if no surrogate variables are specified. This function provides an user with the flexibility of estimating the actual gene-variety interaction effects from a certain ANCOVA model with a specific choice on the number of surrogate variables, which can be selected depending on the complexity of the situation under study. 

•The second function **svpls** calls the first function **fitModel** to fit a number of ANCOVA models (specified by **pmax**) to the data and selects the optimal model as the one with the minimum value of the Akaike’s Information Criterion (AIC) [8]. This model is then used to predict the actual pattern of differential expression of the genes over the two sample varieties by performing a multiple hypothesis testing at specified value of the false discovery rate (FDR) [9] (specified by **fdr**). 

While the Benjamini-Hochberg correction is used by default in our R package the p-values returned by the **svpls** object provides an user with the flexibility of applying several other FDR controlling techniques and also peforming the more specifically targeted gene set enrichment analyses.

**Figure 1:**
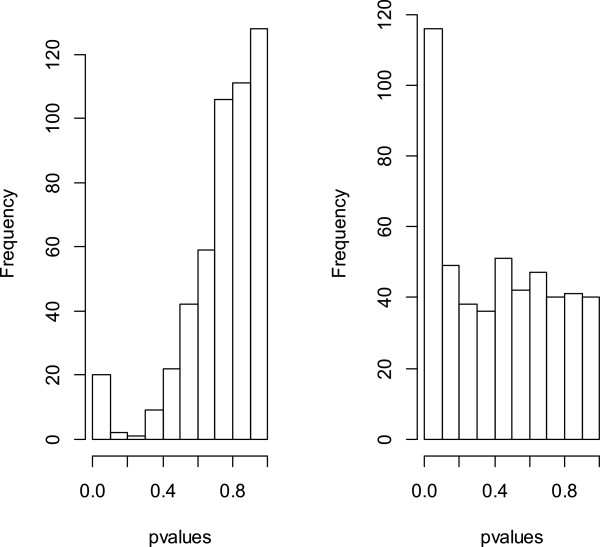
**Histograms of the unadjusted and adjusted p-values.** This figure exhibits two histograms from an analysis of the data **hidden _fac.dat**, one for the unadjusted p-values for testing the variety-based differential gene expression (found from the standard ANOVA model) and the other corresponding to the adjusted p-values obtained after correcting the hidden variability in the data by our R package **svapls**.

We compute p-values from a differential testing of the genes with the estimated effects from standard ANOVA and the optimal ANCOVA model selected by our R package. A side-by-side plot of their corresponding histograms clearly demostrate the efficacy of the function **svpls** in our package in terms of the proximity of the set of larger p-values towards the uniform distribution (Figure 1).

• The third function **hfp** produces a heatmap for the PLS-imputed estimate of the residual expression heterogeneity corresponding to an user-specified set of genes and samples (specified by **gen** and **ind** respectively). This enables us to understand how intensely the latent factors from a certain set of subjects affect the true expression levels of a specified set of genes. 

**Figure 2:**
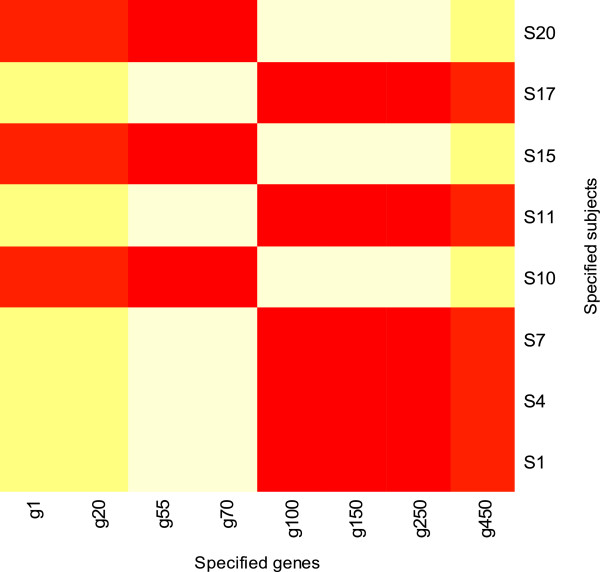
**Heatmap showing the hidden variability in the data hidden_fac.dat owing to the specified set of subjects and genes.**

This produces a plot revealing the way the hidden variable affects the expression pattern of the selected group of genes over the specified subjects (Figure 2). Clearly, we can observe a substantial difference in the expression variability caused by the latent factor for subjects and the rest specified under the selected group.

### Comparative evaluation with other available software packages

In this section we illustrate the application of the R package along with the other three popular software packages through a family of simlulation analyses conducted with two sample sizes 20 and 40 under three different values of the noise-to-signal ratio (*η*=0.05,0.1 and 0.15) controlling the relative intensity of the random error and primary signal variances from low to high [1]. In each simulation study we generate correlated expression measurements on 1000 genes over *k* subjects, (*k*=20,40) classified equally into two groups 1 and 2. Overall, we consider two different settings: (1) The genes are affected by a highly complex subject-specific confounder (mixture of two normal random variables) with a small variance and (2) The genes are affected by two widely different subject-specific confounders (one mixture of two normal random variables and another mixture of two exponential random variables), both with very high variances [1]. Under both the settings, the first 70 genes are considered to be truly differentially expressed over the two varieties while the rest are chosen as non-significant. The simulation study is based on computation of the average values of two right decision indicators (sensitivity, specificity) and two wrong detection indicators (false discovery rate and false non-discovery rate), evaluated from 1000 Monte-Carlo replications (Tables 1 and 2 for setting 1 and 3,4 for setting 2). The obtained results from the two simulation settings clearly reveal the superior sensitivity of **svapls** compared to other three R packages **sam**, **limma** and **sva**, under most of the combinations of group size and noise-to-signal ratios. This illustrates the efficiency of our R package in discovering a higher proportion of the truly significant genes compared to the existing software packages. The sensitivity of **sam** is comparable to our method for a higher sample size under setting 1 (Tables 1 and 2) and is very close or marginally better under setting 2 (Tables 3 and 4), but is adversely impacted by its significantly large false discovery rate. Specifically, the sensitivity obtained from our R package becomes almost similar or slightly better than **sam** as the group size is increased from 10 to 20 (Table 4). Moreover, the specificity rate is the best for **svapls** closely followed by **sva**, while **sam** and **limma** are less efficient in this context. In addition, the average error rates FDR and FNR are much lower for **svapls** compared to the other three software packages. Thus, overall our R package is capable of discovering the truly differentially expressed genes with more power along with an efficient control over the wrong detections (non-detections).

**Table 1:** **Average performance measures from a sensitivity analysis of the simulated gene expression data on 20 subjects (10 being in each group) under setting 1, with the four software packages limma**, **sam**, **sva and svapls**

**Method**	**Sensitivity**	**Specificity**	**FDR**	**FNR**
*η*=0.05				
LIMMA	0.2287	0.6276	0.4285	0.2089
SAM	0.9239	0.6066	0.7278	0.0125
SVA	0.3311	0.9987	0.0475	0.0456
SVAPLS	0.9464	0.9998	0.0023	0.0039
*η*=0.10				
LIMMA	0.2307	0.6566	0.3724	0.2295
SAM	0.8880	0.6410	0.6596	0.0147
SVA	0.2882	0.9988	0.0481	0.0469
SVAPLS	0.9098	0.9994	0.0076	0.0065
*η*=0.15				
LIMMA	0.1956	0.6672	0.3689	0.2164
SAM	0.8522	0.6709	0.6140	0.0193
SVA	0.2474	0.9990	0.0458	0.0485
SVAPLS	0.8660	0.9991	0.0130	0.0097

**Table 2:** **Average performance measures from a sensitivity analysis of the simulated gene expression data on 40 subjects (20 being in each group) under setting 1, with the four software packages limma**, **sam**, **sva and svapls**

**Method**	**Sensitivity**	**Specificity**	**FDR**	**FNR**
*η*=0.05				
LIMMA	0.7863	0.2283	0.7719	0.5037
SAM	0.9793	0.5773	0.7724	0.0033
SVA	0.5659	0.9977	0.0475	0.0311
SVAPLS	0.9954	0.9998	0.0026	0.0003
*η*=0.10				
LIMMA	0.7577	0.2479	0.7432	0.5564
SAM	0.9854	0.6215	0.7055	0.0020
SVA	0.5695	0.9978	0.0471	0.0309
SVAPLS	0.9897	0.9994	0.0083	0.0008
*η*=0.15				
LIMMA	0.7307	0.2389	0.7464	0.5865
SAM	0.9816	0.6448	0.6609	0.0023
SVA	0.5393	0.9980	0.0443	0.0331
SVAPLS	0.9830	0.9990	0.0131	0.0012

**Table 3:** **Average performance measures from a sensitivity analysis of the simulated gene expression data on 20 subjects (10 being in each group) under setting 2, with the four software packages limma**, **sam**, **sva and svapls**

**Method**	**Sensitivity**	**Specificity**	**FDR**	**FNR**
*η*=0.05				
LIMMA	0.2111	0.5367	0.5328	0.2818
SAM	0.6290	0.5879	0.7474	0.0625
SVA	0.0445	0.9998	0.0405	0.0164
SVAPLS	0.6408	0.9998	0.0029	0.0200
*η*=0.10				
LIMMA	0.1953	0.5545	0.4871	0.2942
SAM	0.5580	0.6079	0.7342	0.0606
SVA	0.0239	0.9998	0.0514	0.0148
SVAPLS	0.5125	0.9996	0.0077	0.0263
*η*=0.15				
LIMMA	0.1900	0.5597	0.4838	0.3039
SAM	0.5412	0.6126	0.7363	0.0600
SVA	0.0190	0.9999	0.0395	0.0148
SVAPLS	0.4544	0.9996	0.0112	0.0297

**Table 4:** **Average performance measures from a sensitivity analysis of the simulated gene expression data on 40 subjects (20 being in each group) under setting 2, with the four software packages limma**, **sam**, **sva and svapls**

**Method**	**Sensitivity**	**Specificity**	**FDR**	**FDR**
*η*=0.05				
LIMMA	0.5632	0.1690	0.8399	0.6629
SAM	0.7845	0.6199	0.7158	0.0312
SVA	0.1651	0.9993	0.0469	0.0329
SVAPLS	0.8324	0.9998	0.0024	0.0111
*η*=0.10				
LIMMA	0.5637	0.1640	0.8333	0.6967
SAM	0.7378	0.6507	0.6471	0.0342
SVA	0.1428	0.9994	0.0461	0.0331
SVAPLS	0.7535	0.9995	0.0071	0.0165
*η*=0.15				
LIMMA	0.5660	0.1619	0.8315	0.7183
SAM	0.6983	0.6553	0.6481	0.0372
SVA	0.1082	0.9995	0.0506	0.0350
SVAPLS	0.6806	0.9995	0.0095	0.0211

### Application on the Golub data

**Figure 3:**
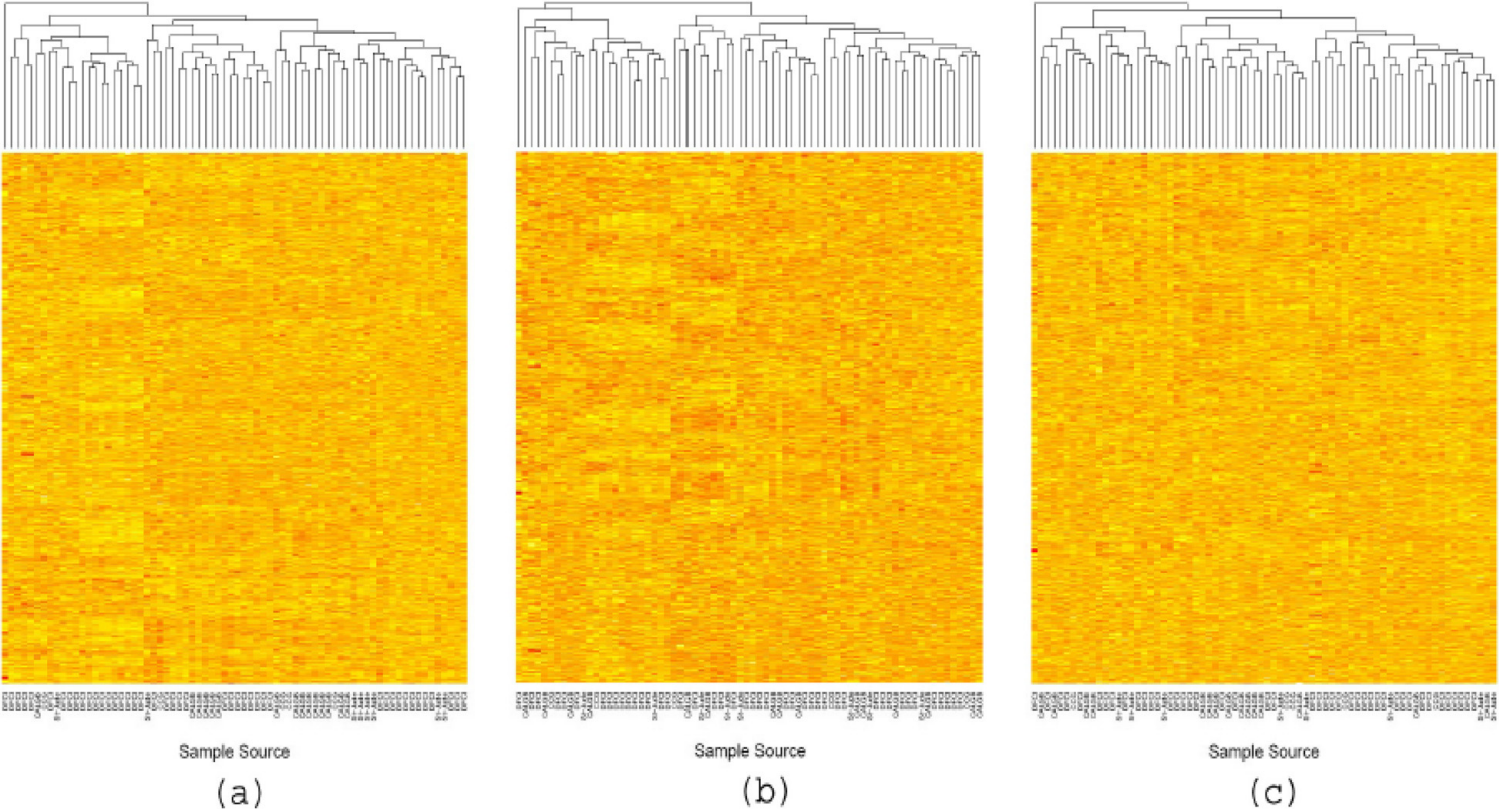
**(a), (b), (c) Heatmaps showing the original and corrected expression levels for the first 1000 genes in the Golub data.(a)** Heatmap for the first 1000 genes in the original Golub expression data. **(b)** Heatmap for the first 1000 genes in the adjusted Golub expression data obtained by use of the R package **ber**. **(c)** Heatmap for the first 1000 genes in the adjusted Golub expression data obtained by the use of our R package **svapls**.

Now, we explore the performance of **svapls** on the pre-processed ALL/AML dataset [10,11]. It contains the log-transformed expression levels of 7129 genes over-two groups of patients: 47 having Acute Lymphoblastic Leukemia (ALL) and 25 suffering from Acute Myeloid Leukemia (AML). The patient tissue samples were obtained from the following four sources: (1) Dana-Farber Cancer Institute (DFCI), (2) St-Jude’s Children’s Research Hospital (ST-Jude), (3) Cancer and Leukemia Group B (CALGB) and (4) Children’s Cancer Study Group (CCG). This inherent classification in the data can potentially generate significant batch effects that may distort the original expression pattern of the genes. This motivated the implementation of our R package on this dataset. The corrected expression matrix for the first 1000 genes returned from the use of the **svpls** function on this data demonstrates that the batch effects due to variability in the sample sources have been removed effectively. The haphazard distribution of the samples from the four sources in the corrected gene expression matrix wipes out the additonal effects owing to the observed batch-specific clustering in the original data. In this context **svapls** fares equally well compared to another popular R package **ber** for removing batch effects in microarray data [12] (Figure 3).

**Figure 4:**
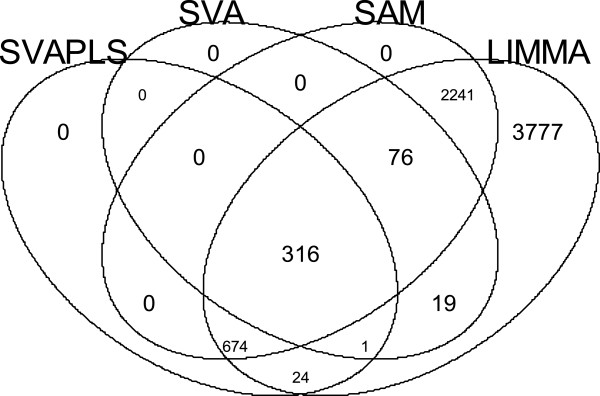
**A Venn-diagram showing the overlap pattern of the genes detected to be significant from the Golub data by svapls**, **sva**, **sam and limma**.

Overall, limma detects 7128 genes followed by 3307 genes from **sam**, 1015 genes from our **svapls** and 412 genes from **sva**. A Venn diagram (Figure 4) represents the extent of overlap between the genes detected by the four softwares. Specifically, **limma** detects all the genes that are found to be significant from the other three softwares. This may be attributable to its high false discovery rate (FDR) as was observed in the simulation study. Interestingly, **svapls** detected 24 genes that are missed by both **sam** as well as **sva**. Among them the genes CD74, TNFRSF1A, LCN2 and GSN deserve special mention. All these genes are either related to some type of cancer or regulate cell growth(or apoptosis). CD74 plays an important role in multiple myeloma and its higher expression induces tumor cell malignancy [13]. An isoform of the tumor necrosis factor TNFRSF1A is associated with the development of Acute Lymphoblastic Leukemia (ALL) in children [14]. Specifically, LCN2 has been found to be connected with Acute Myelogenous Leukemia (AML) [15]. GSN plays a significant role of suppressing tumorigenicity in lung cancer [16] and has a diminuted expression in bladder cancer cells [17].

## Discussion

Various hidden sources of variation are found to exist in a gene expression data that cannot be removed by the standard normalization procedures. But, their effect may be substantial enough to change the expression pattern of the genes over two different varieties of samples. The immediate consequence is a large reduction in the detection power of the testing procedure employed to find the truly significant genes, followed by highly elevated error rates. In this project we discuss the development and usage of an R package **svapls** that can tackle a wide variety of hidden effects in a gene expression analysis and can deliver a more accurate inference on the differential expression variability of the genes between two groups of samples (tissues). We illustrate the superior performance of our R package in comparison to three other popular softwares available for differential gene expression analyses. The high detection power (sensitivity) of **svapls** along with the reasonably small error rates provides it a significantly better edge over the competing softwares. Specifically, **sva** is outperformed by our package in terms of the sensitivity (power), while **sam** comes close and performs marginally better in some cases, although its competence is severely marred by the considerably high false discovery rate (FDR) and substantially low specificity rate. In addition the graphical representation of the hidden variation (by the function **hfp**) from our package enables the user to understand the pattern in which the hidden sources of variability affect the expression signals of any specified subset of genes over a selected group of subjects/samples. This paves the way to more sophisticated analyses of subject-set specific gene expression variability in the data. Application of our package on the Golub data demonstrates its efficacy in removing the significant batch effects from the collected/analyzed samples. Moreover our package detects four additional genes (missed by both **sva** and **sam**) that have been found to be connected to Leukemia or some other type of cancer.

## Conclusions

The R package **svapls** can detect a wide variety of hidden factors in a gene expression study and adjust for them appropriately, in order to provide a more accurate inference on the expression pattern of the genes between two different types of tissues. In particular, the superior detection power and small error rate gives our R package a substantially better edge over the competing softwares considered in the analysis.

## Availability and requirements

### Project name

R package**svapls**

### Project home page

http://cran.rproject.org/web/packages/svapls/index.html

### Operating system and R version

The R package is platform independent and is compatible with all the versions of R same as or higher than 2.0.

### License

GPL-3

## Competing interests

The authors declare that they have no competing interests.

## Authors’ contributions

SC wrote the R package including its evaluation and drafted the manuscript. SD and SD contributed to the structure of the simulation studies, application and planning of the manuscript. All authors read and approved the final manuscript.
